# Occurrence of Potentially Toxic Elements (PTEs) Coupled with Mineralogical and Morphological Characteristics of Residential Indoor Vacuum Dusts from the City of Thessaloniki, Northern Greece

**DOI:** 10.3390/toxics14040306

**Published:** 2026-03-31

**Authors:** Christina Kotsakostoudi, Anna Bourliva, Lambrini Papadopoulou, Nikolaos Kantiranis

**Affiliations:** 1 Department of Mineralogy-Petrology-Economic Geology, School of Geology, Aristotle University of Thessaloniki, 54124 Thessaloniki, Greece; kchristina91@gmail.com (C.K.); lambrini@geo.auth.gr (L.P.); kantira@geo.auth.gr (N.K.); 2 Soil Science Laboratory, School of Agriculture, Faculty of Agriculture, Forestry and Natural Environment, Aristotle University of Thessaloniki, 54124 Thessaloniki, Greece

**Keywords:** indoor dust, PTEs, contamination indices, health risk, Thessaloniki

## Abstract

This study investigates the occurrence, sources, and health risks of PTEs in residential vacuum dusts from the city of Thessaloniki, Greece. A total of 20 dust samples were collected and analyzed for their chemical, mineralogical, and morphological characteristics using pXRF, XRD, and SEM-EDS techniques. The results revealed elevated concentrations of Zn (623 mg kg^−1^), Mn (392 mg kg^−1^), Cu (204 mg kg^−1^), and Cr (185 mg kg^−1^) exceeding crustal averages and global urban soil baselines. Notably, Cr and Mn levels were among the highest recorded for non-industrial urban settings. Source apportionment identified distinct geogenic and anthropogenic contributors, including construction materials, outdoor soil resuspension, and indoor alloy-related sources such as stainless steel and soldering components. Health risk assessment based on USEPA models showed ingestion as the dominant exposure route, particularly for children. Chromium and As were identified as the main non-carcinogenic and carcinogenic contributors, with children’s hazard index (HI) values exceeding safety thresholds (HI = 1.04) in some cases. The cancer risk (CR) for Cr ranged from 2.49 × 10^−5^ to 6.55 × 10^−5,^ not exceeding the acceptable limit (10^−4^). The findings highlight the multifaceted nature of indoor dust contamination in urban environments and underscore the need for continued monitoring and targeted mitigation to protect vulnerable populations.

## 1. Introduction

House dust (HD), a common environmental contaminant in indoor environments, is a complex mixture of particles that can be extremely harmful to human health [1,2]. It is a significant vector for the spread of several potentially harmful compounds, including heavy metals, flame retardants, phthalates, pesticides, and allergens [3,4,5,6,7]. Indoor dust particles are produced from various sources, including soil intrusion, human activity, the use of household products, and the deterioration of building materials, and can vary greatly in composition based on geographical location, socioeconomic factors, and lifestyle [8]. Numerous studies conducted over the years have demonstrated that house dust contains a variety of potentially toxic elements (PTEs), as well as mineralogical and morphological particulates that can cause a number of health problems, especially in susceptible groups like children, the elderly, and people with underlying respiratory disorders [9,10,11].

Among indoor contaminants, PTEs such as lead (Pb), arsenic (As), cadmium (Cd), mercury (Hg), and chromium (Cr) are particularly concerning because of their systemic toxicity, persistence, and capacity for bioaccumulation [12,13]. In this study, the term potentially toxic elements (PTEs) is used as a broad environmental category that encompasses both elements that are inherently toxic even at low concentrations, as well as elements whose toxicity is concentration-dependent. The long-term health effects of these substances, especially in young children who are disproportionately vulnerable due to behavioral and physiological factors, have raised concerns about their presence in household dust [14,15,16]. Long-term indoor dust exposure can lead to pollutant exposure levels up to 1000 times higher than outdoor exposure [17] and is associated with serious health consequences. According to the World Health Organization [18], indoor air pollution is responsible for 2.9 million deaths per year, including lung cancer, ischemic heart disease, pneumonia, stroke, and chronic obstructive pulmonary disease. For instance, Pb has been found to be one of the most common and dangerous metals in household dust, especially in residences constructed prior to the 1970s, when Pb-based paints were widely used. According to studies, children who are exposed to Pb from household dust may experience behavioral issues, cognitive impairments, and developmental delays [19]. The U.S. Environmental Protection Agency notably tightened its residential dust Pb hazard standards in response to this [20]. Furthermore, arsenic, a well-known carcinogen, is frequently found in household dust because it is widely used in wood preservatives and is detected in contaminated groundwater. Results from both epidemiological studies and experimental research have shown that long-term exposure to As increases the risk of developing both cancerous and non-cancerous conditions, including immune system toxicity [21,22].

In addition to PTEs, a variety of mineralogical particles are found in house dust, whose presence and makeup vary depending on a number of factors, such as the home’s location, the materials used in its construction, and the effects of the outdoor environment [23]. Though the chemical composition and toxicology of household dust have been the main objective of many studies, there are still few mineralogical studies available, with the majority of earlier scientific works ignoring the mineral hosts. Our comprehension of how particular mineral phases affect the behavior, mobility, and bioavailability of PTEs in indoor environments is hampered by this lack of data. However, numerous minerals, such as quartz, feldspars, mica, clay minerals, and carbonates, have been detected in the few studies that have been conducted on the mineralogy of household dust [23,24,25]. These minerals can differ in relative abundance; dust from outdoor sources tends to contain quartz and clay minerals, whereas dust from indoor sources, like decaying building materials, tends to contain carbonates and silicates. Because they can cause respiratory illnesses, especially when inhaled as fine particles, mineral dusts are of particular concern. For example, inhalation of crystalline silica (e.g., quartz) is known to increase the risk of developing lung cancer and other chronic respiratory conditions like silicosis [26,27,28].

Another crucial factor to take into account when assessing the health risks associated with dust particles is their morphology. Particles’ capacity to enter the respiratory system, accumulate in the lungs, and cause inflammatory reactions can be strongly influenced by their size, shape, and surface area [29]. Studies of house dust particles using scanning electron microscopy (SEM) and transmission electron microscopy (TEM) have shown that the shapes of particles greatly vary, with both spherical and irregularly shaped particles being detected [30,31,32,33,34]. Irregularly shaped particles have a greater surface area, which raises their potential for chemical reactivity and bioaccumulation of harmful substances, whereas spherical particles have a tendency to settle quickly [35]. The risk that these particles pose to human health is further increased by the fact that their surface characteristics, such as their charge and roughness, are crucial in their capacity to adsorb harmful substances like heavy metals and organic pollutants.

The health risks associated with the inhalation, ingestion, or dermal contact of PTEs and mineral particles in house dust are multifaceted. Chronic exposure to dust-borne heavy metals, such as Cr, Pb, and As, has been linked to developmental and neurological disorders, particularly in children [14,15,16]. Children are particularly vulnerable due to their higher dust ingestion rates and developing immune and respiratory systems. Evidence from studies on dust contamination has indicated that young children living in homes with high levels of toxic dust components are at an increased risk of developmental delays, asthma, and other respiratory conditions [36]. Moreover, the ubiquitous nature of household dust and the frequent indoor exposure present unique challenges for environmental health research, as the cumulative and long-term effects of low-dose exposure may not become apparent for many years.

In the past, most research on indoor dust contamination has been conducted using a variety of methodologies and focused on localized case studies with a narrow spatial scope [37,38,39]. But more thorough evaluations are now available thanks to the steady emergence of larger national and international surveys. Notable examples include large-scale studies conducted in the UK [40], Germany [41], Canada [42], and China [43], which collectively expanded the understanding of indoor contaminant patterns across diverse settings. For example, Thornton et al. [40] conducted one of the earliest country-wide assessments in the UK, analyzing Pb concentrations in 97 homes and correlating dust Pb loading with blood Pb levels in children, while Rasmussen et al. [42] launched a nationwide house dust study, remediating Pb bioaccessibility and speciation across diverse provinces. The most ambitious and globally coordinated effort to date is the DustSafe program (https://www.360dustanalysis.com/), which has yielded a valuable dataset by utilizing standardized sampling and analytical protocols to assess indoor dust composition across 35 countries, including Greece. This large-scale citizen science project enabled consistent comparison of trace elements and contaminants in household dust at an international level, addressing the longstanding challenge of methodological variability that has historically limited cross-regional assessments of indoor environments [8].

Studies on the chemical composition and sources of house dust in Greece remain relatively limited, despite growing recognition of indoor dust as a major pathway of human exposure to PTEs. Existing research has focused on specific urban and industrial areas such as Athens [44,45], Volos [46], and Stratoni [47], revealing elevated concentrations of Pb, Zn, Cu, and Cr, often linked to traffic emissions, industrial activity, and indoor sources. However, the PTE contents in household dust from the city of Thessaloniki have not been studied yet. Additionally, previous studies focused largely on elemental concentrations, while mineralogical and morphological characteristics that are essential in order to understand the behavior, sources, and potential health impacts of dust particles are quite limited. The present study aimed to provide a comprehensive assessment of indoor house dust by integrating multiple analytical and interpretive approaches. This interdisciplinary approach is a novel contribution to the existing indoor environmental studies in Greece, providing new insights regarding the composition and potential risks of household dust. Specifically, the objectives were: (i) to perform chemical analysis of potentially toxic elements (PTEs) in house dust samples to determine their concentration levels; (ii) to evaluate the degree of contamination and classify the pollution levels; (iii) to apply source apportionment techniques, in order to identify and distinguish between geogenic and anthropogenic sources of these elements; and (iv) to conduct a detailed health risk assessment, estimating both non-carcinogenic and carcinogenic risks for adults and children. By incorporating mineralogical and morphological profiling alongside elemental data, this study contributes additional insight into the physical and chemical heterogeneity of house dust.

## 2. Materials and Methods

### 2.1. Study Area and Sampling

Thessaloniki (40.61° N, 22.91° E) is the second-largest city of Greece, with approximately 1.1 million inhabitants residing in the broader metropolitan area, according to the most recent national census conducted in 2011. The city is located in the innermost part of the Thermaikos Gulf and is surrounded by Mount Hortiatis to the north and northeast, which rises to an altitude of 1200 m. Several stable residential communities are situated in the wider area, while an extensive industrial zone lies to the northwest, comprising both small- and large-scale industrial activities such as oil refining, petrochemical production, fertilizer and cement manufacturing, non-ferrous metal smelting, iron and steel manufacturing, truck and auto painting, metal recovery facilities, electrolytic MnO_2_ production, anodized aluminum manufacturing, scrap metal incineration, tire production, and lubricating oil recovery [48].

According to Manoli et al. [49], Thessaloniki’s climate is typically Mediterranean and is strongly influenced by the sea breeze. The mean monthly relative humidity ranges from 47% to 80%, with an average annual precipitation of 490 mm. Temperatures vary from 5.5 °C in January to 28 °C in August. The prevailing wind directions are from the north/northwest (25%) and south/southwest (30%), while calm conditions occur approximately 20% of the time. These meteorological conditions, combined with the complex topography and urbanization, contribute to the limited dispersion of atmospheric pollutants, leading to their accumulation in both outdoor and indoor environments, such as household dust.

A total of 20 samples of residential indoor dust were collected from selected residences in the city of Thessaloniki. Particular emphasis was given to achieve a spatially uniform sampling, ensuring an even geographic distribution of the selected residences within the densely built urban area of Thessaloniki. Even though the number of the sampled dusts may be considered relatively limited, similar sample sizes have been adopted in previous studies [46]. Precise residential locations were not unveiled so as to assure confidentiality. However, the selected households allow a representative assessment of indoor dust characteristics across the city of Thessaloniki. Information on household characteristics and activities was obtained, indicating that about 38% of the sampled houses were moderately sized (about 70 m^2^) and had at least one smoker. There were roughly 20–30-year-olds and were distributed across different floor levels, reflecting variability in their vertical position within the urban setting. In addition, they were occupied by 1 to 3 individuals, and half of the sampled households reported that they were wearing shoes inside the house.

Sampling took place in June 2024, prior to the onset of the rainy season, and spanned a two-month period. The house dust samples were collected by the residents themselves, who vacuumed hard floor surfaces in all living spaces of their homes multiple times. The vacuum bags, which were newly installed prior to sampling, were voluntarily provided. Upon collection, the bags were sealed, transported to the laboratory, and processed for further analysis. Dust was carefully removed from the vacuum bags, homogenized, while particular attention was given so as to fully recover the fine particles adhering to the bag material by gently brushing the inner surfaces. Though minor losses cannot be excluded, they are considered negligible and are expected not to significantly influence the overall results. The entire content of each bag was air-dried and sieved using a 500 μm mesh to remove coarse particles. Subsequently, a 150 μm mesh sieve was used to obtain the final <150 μm particle size fraction, which was retained for further laboratory analyses. The <150 μm particle size fraction, which encompasses a broad spectrum of particle sizes including finer fractions, was selected for analysis as it is considered representative for human exposure to indoor dust, particularly due to its higher contaminant loadings, better homogeneity compared to coarser fractions, and greater potential for adherence to hands [50,51]. Although the <150 μm fraction does not isolate finer particles, it serves as a composite proxy that includes them and is therefore commonly used in exposure and risk assessment studies [8,42]. Each bag was handled carefully to avoid cross-contamination of the samples.

### 2.2. Analytical Methods

To identify the basic properties affecting the mobility and bioavailability of heavy metals, a variety of physicochemical characterizations were performed on all indoor dust samples. The mineralogical composition was determined using a Bruker D8 Advance (Bruker Corporation, Billerica, Massachusetts) diffractometer equipped with a DaVinci goniometer, a LynxEyeXeT (1D mode) silicon strip detector, a 17 mm divergence slit, and an 18 mm anti-scatter slit. CuKα1 radiation was used (Ni filter, 40 kV, 40 mA). Scans were performed on randomly oriented powder samples (0.5 g) over the 3–63° 2θ range, with a step size of 0.019° 2θ and a counting time of 0.25 s per step. Data were processed using EVA v7.1^®^ software with the ICDD PDF2-2023 database. Semi-quantitative phase analysis was carried out using the Rietveld refinement method with TOPAS 6.0^®^ software (2016), based on model patterns of the crystalline structures of each identified mineral phase, optimized using a non-linear least squares approach. Amorphous components of indoor dust were not quantified in the present study.

The morphology of the house dust particles, as well as representative microanalyses at selected points, were examined using a JEOL JSM-6390LV scanning electron microscope (SEM) (Tokyo, Japan) equipped with an Oxford INCA 300 energy-dispersive spectrometer (EDS) (Abingdon, UK). A pure cobalt sample was used as a calibration standard. Analysis conditions included an accelerating voltage of 20 kV, beam current of 0.4 mA, analysis time of 80 s, and an electron beam diameter of approximately 1 μm. To ensure electrical conductivity, a conductive coating was applied via carbon evaporation in a vacuum using an Agar Carbon Coater. The carbon film thickness did not exceed 200 Å in order to ensure optimal conductivity without compromising the instrument’s sensitivity. SEM analysis was performed on selected household dust samples. Representative dust material was mounted on stubs and placed into the SEM chamber for analysis.

Elemental concentrations were determined via pXRF. Each sample was placed in a standard sample cup, which was fitted with a thin X-ray transparent film in order to ensure a flat and uniform analytical surface. Special emphasis was given to sufficient sample thickness so as to minimize matrix and substrate effects on X-ray signal intensity. A Bruker S1 Titan 600 (Bruker Corporation, Billerica, MA, USA) with a 4 W Rh X-ray tube, 5 mm spot size, a silicon drift detector (resolution < 145 eV), and the inbuilt Geoexploration mode, a factory calibration method for soils, was used for pXRF analysis. Three energy ranges (15, 30, and 50 kV) were used for the analysis, which lasted 30 s each for a total of 90 s. Quality assurance and quality control procedures ensured the reliability of the pXRF measurements. Analytical triplicates yielded relative standard deviations below <10%. Certified reference materials (CRMs) for both major and trace elements were used to calibrate the elemental contents determined using the Geoexploration mode; calibration curves with R^2^ values greater than 0.9 were produced. Recovery rates and limits of detection (LoD) are provided in Table S1 and Table S2, respectively.

This research does not involve human participants or clinical interventions; therefore, a clinical trial registration number is not applicable.

### 2.3. Contamination Indices

This study used pollution and risk assessment indices to thoroughly evaluate the degree and possible consequences of heavy metal contamination in indoor dust. Indoor dust is a complex and heterogeneous matrix that is composed of both geogenic and anthropogenic materials. Therefore, the application of the following indices, which were originally developed for soils and sediments, should be interpreted with caution.

#### 2.3.1. Enrichment Factor (EF)

A widely used method for assessing the degree of metal contamination and determining possible human contributions is the Enrichment Factor (EF) [52,53]. Metal concentrations are normalized against a reference element, usually Fe, Al, Ti, or Mn, which is considered to be primarily of crustal origin, in order to account for natural variability in sample composition. This method lessens the impact of sample heterogeneity. The EF is calculated using the following formula:

(1) $$EF=\frac{{\left(C_{n}/C_{ref}\right)}_{sample}}{{\left(B_{n}/B_{ref}\right)}_{background}}$$

where C_n_ and B_n_ are the concentrations of the target element in the sample and background, respectively, while C_ref_ and B_ref_ are the concentrations of the reference (normalizing) element in the sample and background, respectively. The selection of the reference element is crucial and is usually determined by its relative abundance and low environmental variability, which guarantees that small variations or geochemical interactions will not have a big impact on the outcomes. Aluminum (Al), which has been used in previous investigations [54,55], was chosen as the reference element for EF calculations in this study due to its comparatively low variability and lower measured concentrations in comparison to the background values, consistent with its use in prior studies. Due to the absence of local background soil values, which could provide a more site-specific baseline, average abundances of upper continental crust [56] were used as background values in line with previous studies on indoor dust [8]. EF values greater than 10 typically suggest substantial anthropogenic input, whereas values below 2 are generally attributed to natural sources [57]. Moreover, EFs are instrumental in assessing the degree of contamination, with classification into five categories: EF < 2 indicates minimal or no enrichment; EF = 2–5, moderate enrichment; EF = 5–20, significant enrichment; EF = 20–40, very high enrichment; and EF > 40, extremely high enrichment [55].

#### 2.3.2. Geoaccumulation Index (I_geo_)

The Geo-accumulation Index (I_geo_), originally proposed by Müller [58], was calculated using the following formula:

(2) $$I_{geo}={log}_{2}\left(\frac{C_{n}}{1.5\times B_{n}}\right)$$

In this equation, C_n_ represents the measured concentration of the element *n* in the household dust samples, B_n_ denotes the geochemical background concentration of the same element, and 1.5 acts as a correction factor to take into consideration lithological variables that naturally create variations in the background matrix. According to I_geo_ values the urban soils are characterized as follows: I_geo_ ≤ 0 indicates uncontaminated, 0–1 indicates uncontaminated to moderately contaminated, 1–2 moderately contaminated, 2–3 moderately to heavily contaminated, 3–4 heavily contaminated, 4–5 heavily to extremely contaminated, and I_geo_ > 5 indicates extremely contaminated dust samples.

### 2.4. Health Risk Assessment

The possible non-carcinogenic and carcinogenic health effects for adults and children resulting from exposure to PTEs in household dusts were evaluated using the risk assessment models developed by the United States Environmental Protection Agency [59]. The three main routes of human exposure to impacted HD particles, i.e., direct ingestion, inhalation, and dermal contact, were assessed. The average daily doses (ADD) (mg kg^−1^ d^−1^) of selected PTEs (those exhibiting mean EF > 2) across these exposure pathways were determined based on standard equations provided by the USEPA [60], as follows:

(3) $${ADD}_{ing}=\frac{C_{dust}\times IngR\times EF\times ED\times CF}{BW\times TA}$$

(4) $${ADD}_{inh}=\frac{C_{dust}\times InhR\times EF\times ED}{PEF\times BW\times TA}$$

(5) $${ADD}_{derm}=\frac{C_{dust}\times SA\times AF\times ABS\times EF\times ED\times CF}{BW\times TA}$$

where ADD_ing_, ADD_inh_, and ADD_derm_ are the daily doses of exposure to elements in HDs through ingestion, inhalation, and dermal contact, respectively. The parameters utilized in the models for children and adults, which were taken from the USEPA Risk Assessment Guidance, are listed in Table S3.

The non-carcinogenic and carcinogenic health risks were evaluated using the Hazard Quotient (HQ), cumulative Hazard Index (HI), and carcinogenic risk (CR) indices. Non-carcinogenic risks via ingestion (HQ_ing_), inhalation (HQ_inh_), and dermal contact (HQ_derm_) were calculated following the equations:

(6) $${HQ}_{ing}=\frac{{ADD}_{ing}}{{R_{f}D}_{ing}}$$

(7) $${HQ}_{inh}=\frac{{ADD}_{inh}}{{R_{f}D}_{inh}}$$

(8) $${HQ}_{derm}=\frac{{ADD}_{derm}}{{R_{f}D}_{derm}}$$

In this context, R_f_D_ing_ refers to the oral reference dose (mg kg^−1^ day^−1^), R_f_D_inh_ refers to the inhalation reference dose (mg kg^−1^ day^−1^), and R_f_D_derm_ indicates the dermal reference dose (mg kg^−1^ day^−1^). These values denote the highest daily intake that is permitted via ingestion, inhalation, and dermal contact, respectively, that is not anticipated to have detrimental impacts or detrimental health outcomes in humans over a lifetime. Hazard quotient (HQ) values exceeding the safety threshold of 1 indicate a potential risk of adverse health consequences [59].

The hazard index (HI) represents the sum of HQs, and it is calculated as:

(9) HI=∑HQi

where i defines the different exposure pathways that were previously stated. HI < 1 means no risk of non-carcinogenic effects, whereas HI > 1 indicates a probability of adverse health effects, which increase with the increase in the health index.

The carcinogenic risk (CR) reflects the probability of an individual developing cancer at some point in their lifetime due to long-term exposure to a carcinogenic substance, and it is calculated using the following formula:

(10) $$CR={ADD}_{i}\times {SF}_{i}$$

Once more, “i” denotes the individual exposure pathways in this evaluation, while SF_i_ stands for the slope factor (mg kg^−1^ d^−1^) connected to each route. In terms of the probability of cancer development, the carcinogenic risk (CR) is considered insignificant if it is below 10^−6^. On the other hand, a CR score above 10^−4^ suggests a possibly increased cancer risk. Values in the range of 10^−6^ to 10^−4^ are generally considered to be an acceptable or tolerable range of risk for human and public health [50]. The R_f_Ds and SF_i_ for each metal are based on the USEPA Regional Screening Level (RSL) tables [61] and are presented in Table S4. The carcinogenic risk for Cr was calculated using the available oral SF for Cr(VI) based on total Cr concentrations, as Cr speciation data were not determined in the study. This approach of risk estimation, associated with assuming that total Cr is present as Cr(VI), results in overestimation of carcinogenic risk, so the reported CRs should be interpreted with caution.

### 2.5. Statistics Analysis

Multivariate statistical analyses, including Principal Component Analysis (PCA) and Hierarchical Cluster Analysis (HCA), were utilized. PCA, performed on the dataset of elemental concentrations, was carried out using Varimax rotation to make interpretation easier by focusing variable loadings on fewer factors, keeping just those with eigenvalues greater than 1. The Kaiser-Meyer-Olkin (KMO) measure of sampling adequacy, which was 0.743, and Bartlett’s Test of Sphericity, which resulted in a chi-square value of 1134.5 (df = 28, *p* < 0.001), validated the data’s eligibility for PCA by showing enough correlations among variables. HCA, employed as an exploratory tool to identify grouping patterns among variables, was carried out using Ward’s linkage approach, and squared Euclidean distance was used to measure similarity. Prior to clustering, the data were converted to z-scores, and the outcomes were displayed in a dendrogram. Moreover, all statistical analyses were performed using SPSS software (version 29.0, IBM Corp., Armonk, NY, USA).

## 3. Results

### 3.1. Mineralogy of House Dust Samples

Summary statistics of the mineralogical composition of the crystalline fraction of the studied HDs are presented in Table 1. Mineralogical characterization of household dusts revealed the predominance of common constituents of high crystallinity, such as calcite and quartz, which were consistently detected across all analyzed samples in line with previous studies [62]. Specifically, calcite was detected in concentrations ranging from 27 to 60 wt% with soil particles detected indoors, along with building materials such as remnants of concrete, plaster, or tiles being its most probable sources in the household environment [23]. Quartz was detected in concentrations ranging from 12 to 31 wt%, with the potential health hazards associated with crystalline SiO_2_ being investigated by numerous researchers [27,28]. Moreover, plagioclases (primarily albite), dolomite, talc, micas, and halite were identified at lower concentrations and were present in the vast majority of analyzed samples. Swelling clay minerals were also identified with concentrations ranging from 2 to 7 wt%. On the other hand, cristobalite and K-feldspars (mainly orthoclase) were detected in minor amounts (1–3 wt%) only in dust samples from households located near the city center of Thessaloniki, while hematite was identified in a minor amount of the sampled house dusts, in concentrations between 1 and 2 wt%.

Though the lack of extensive studies on the mineralogy of household dusts, our findings align with the limited mineralogical characterizations reported by Marinho-Reis et al. [23], who identified similar phases in urban indoor dust from private homes of a Portuguese industrial city and Yang et al. [25] in household dusts from Beijing, China. Finally, our findings are in agreement with our prior research [63] on the mineralogical composition of road dust in the city of Thessaloniki, suggesting a potential infiltration of traffic-related particles into the indoor environment.

### 3.2. Morphology of House Dust Particles

**Figure 1:**
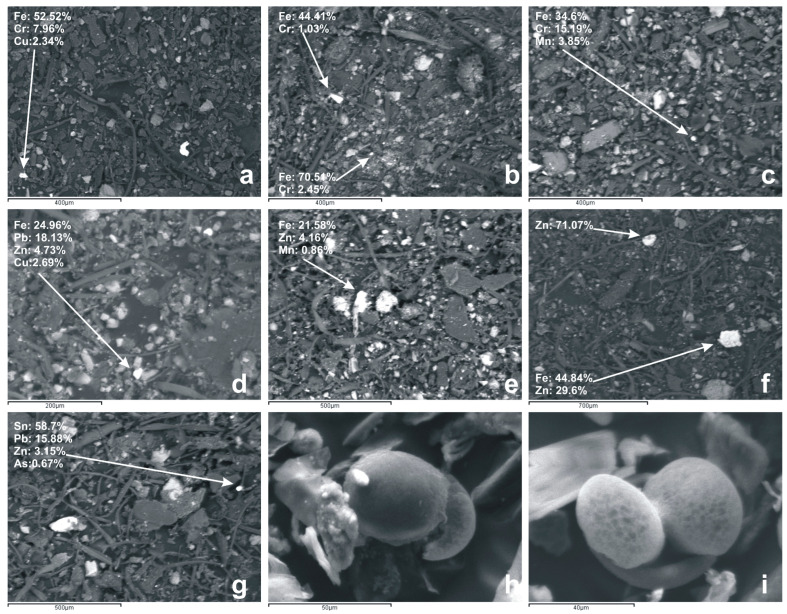
Scanning electron microscopy (SEM) images of particles detected in house dusts from the city of Thessaloniki, Greece. (**a**–**c**) Fe/Cr-containing particles probably originating from stainless steel corrosion. (**d**–**f**) Fe/Zn-containing particles sourced from outdoor activities (**g**), Sn/Pb-rich particles probably originating from soldering alloys used in electronic components and assemblies (**h**,**i**), spherical organic particles attributed to pollen grains.

The morphological examination of the analyzed house dust samples from the city of Thessaloniki revealed that house dust particles appear in angular, irregular, and spherical shapes, as well as in irregular agglomerates, as presented in Figure 1. Analytical observations revealed the dominance of aluminosilicates, along with particles rich in Ca- and Fe. According to previous studies on mineral dust in indoor settings, the aluminosilicate particles revealed angular or irregular morphologies, which are typical of their natural origin [64,65]. In line with findings from other researchers [66], the Ca-rich particles are primarily identified as calcite or irregular agglomerates, indicating a likely origin from the deterioration of indoor flooring or construction materials inside the home.

Additionally, Fe-rich dust particles were detected, displaying irregular and angular morphologies and having iron levels as high as 70%. These Fe-bearing go hand in hand with higher amounts of PTEs like Cr, Cu, Ni, Pb, and Zn (Figure 1), a pattern also noticed in previous studies of indoor particulate matter [30,31].

The Fe/Cr-containing particles (Figure 1a–c), which represented the majority in this study, are likely associated with multiple sources. A significant proportion may originate from natural geogenic sources, as Fe and Cr commonly co-occur in certain rock types and soil minerals, especially in ultramafic or mafic lithologies or even traffic-related emissions, with Fe and Cr being among the most prominent elements identified in urban dust and non-exhaust emissions [35,67,68]. Windborne dust and resuspension of local soils or road dust can introduce Fe–Cr-rich particles into indoor environments, particularly in urban areas with high atmospheric particulate load or poor ventilation. Nevertheless, indoor stainless-steel sources cannot be excluded. Stainless steel is an iron-based alloy that contains primarily Cr in proportions often exceeding 10.5%. Depending on the stainless steel type, additional alloying elements such as Cu and Ni are also present in smaller quantities [69]. Therefore, particles detected in this study that primarily consist of Fe and secondarily of Cr, Cu, and Ni may also originate from stainless steel corrosion, aligning with previous identifications of metal alloy-derived dust indoors [70].

On the other hand, particles composed of Fe/Zn (Figure 1d–f) are likely sourced from outdoor environments, as this alloy is used in the automotive industry to prevent vehicle corrosion [71]. Moreover, brake and tire wear are also known to release Fe- and Zn-enriched particles [72], and several studies have reported co-enrichment of Fe and Zn in indoor dust samples near urban and high-traffic areas, supporting their attribution to non-exhaust traffic emissions. Sn–Pb-rich particles (Figure 1g) may derive from soldering alloys used in electronic components and assemblies, which traditionally utilize Sn–Pb mixtures [73]. Fe–Pb alloys, known for their properties such as high magnetic saturation, high permeability, and strong absorption capacity, are often used in applications like high-performance electromagnets and transformers [74], suggesting that such particles may originate from household or external devices containing these materials.

Lastly, spherical organic particles were also noted and identified as pollen grains (Figure 1h,i), which are also seen in other areas’ indoor environments [25,75].

### 3.3. Elementary Composition of House Dusts

The elemental composition of household dust offers important information about the quality of the indoor environment and possible health risks from extended exposure. Descriptive statistics of both major and potentially toxic elements found in the house dust samples are summarized in Table 2. As can be seen, the analysis of house dust samples showed that the concentrations of major and trace elements varied, and that there were notable variations among the sampled houses, indicating a variety of contamination sources and indoor environmental conditions.

Due to inputs from outdoor sources, construction materials, and urban dust resuspension, Ca and Si had the highest concentrations among the major elements analyzed (mean values: 10.31 ± 3.85 wt% and 4.10 ± 1.07 wt%, respectively) [76]. With average concentrations of 1.38 ± 0.35 wt% and 1.07 ± 0.18 wt%, respectively, Iron (Fe) and aluminum (Al) were also significant. Fe and Al levels are primarily influenced by outdoor sources along with indoor sources and the presence of Fe or Al-containing household materials [77]. Magnesium (Mg), potassium (K), and titanium (Ti) exhibited mean values less than 1%.

Regarding PTEs analyzed, Zn (623 mg kg^−1^) was identified as the dominant pollutant in line with previous studies [34,78,79,80,81], followed by Mn (392 mg kg^−1^), Cu (204 mg kg^−1^), and Cr (185 mg kg^−1^). Specifically, the elemental contents exhibited ranges of 4–11 mg kg^−1^ for As, 75–312 mg kg^−1^ for Ba, 106–279 mg kg^−1^ for Cr, 62–614 mg kg^−1^ for Cu, 263–544 mg kg^−1^ for Mn, 23–160 mg kg^−1^ for Ni, 22–77 mg kg^−1^ for Pb, 11–20 mg kg^−1^ for Rb, 93–479 mg kg^−1^ for Sr, 6–58 mg kg^−1^ for Y, and 339–885 mg kg^−1^ for Zn. The mean concentrations decreased in the order Zn > Mn > Cu > Cr > Sr > Ba > Zr > Ni > Pb > Rb > Y > As. Compared with Earth’s crust abundances [56], the mean concentrations of Zn, Cu, Pb, Cr, As, and Ni were approximately 9.3, 7.3, 3.0, 2.0, 1.7, and 1.4 times higher, respectively, indicating that the sampled house dusts were influenced by human activities. In addition, the sampled house dusts were surpassing the typical abundances reported for worldwide urban soils [82] with mean contents of Cu, Mn, Cr, and Ni being 5.2, 3.9, 2.3, and 2.0 times higher. The obtained CVs (coefficients of variance) were above 30% for nearly all PTEs, indicating high variability and possibly a significant impact from human activity [83,84,85]. Differences among the sampled residences highlighted the complex composition of HD and its diverse sources. They also suggest that indoor and outdoor activities, along with household materials, play a key role in shaping PTE levels.

**Table 2:** Descriptive statistics of major and trace element concentrations in house dust samples from Thessaloniki city, along with reference values.

Element	Min	Max	Mean	Median	SD	Skewness	Kurtosis	CV	Earth’s Crust ^1^	Urban Soils ^2^	Median of 35 Countries ^3^
Ca	5.51	19.69	10.31	10.03	3.85	1.679	4.017	0.373			
Si	2.56	5.65	4.10	4.28	1.07	−0.095	−1.362	0.260			
Al	0.66	1.34	1.02	1.03	0.22	0.014	−0.627	0.211			
Fe	0.68	1.86	1.38	1.45	0.35	−0.764	0.522	0.252			
Mg	0.60	1.08	0.76	0.70	0.16	0.956	0.218	0.205			
K	0.55	1.18	0.80	0.82	0.19	0.338	0.300	0.242			
Ti	0.11	0.32	0.21	0.20	0.06	0.421	1.115	0.271			
As	4	11	8.1	9	2.5	−0.354	−1.127	0.305	4.8	15.9	13.3
Ba	75	312	174.8	159	84.8	0.873	−0.499	0.485	628	853.12	
Cr	106	279	185.4	179	48.6	0.396	0.625	0.262	92	80	86
Cu	62	614	203.9	165.5	157.5	2.257	5.976	0.772	28	39	176
Mn	263	544	391.7	384	92.0	0.422	−0.165	0.235	1000	729	257
Ni	23	160	65.8	50	43.3	1.529	2.060	0.658	47	33	39
Pb	22	77	51.3	52	20.7	−0.213	−1.577	0.403	17	54.5	94
Rb	11	20	14.8	14	3.3	0.360	−1.391	0.223	112	58	
Sr	93	479	175.1	145.5	110.9	2.741	8.110	0.633	320	458	
Y	6	58	13.4	7	16.8	2.937	8.717	1.250	21	23.4	
Zn	339	885	623.0	650	196.5	−0.210	−1.257	0.315	67	158	1110
Zr	0	288	113.4	113.5	82.6	0.760	1.218	0.729	193	255.6	

^1^ [56], ^2^ [82], ^3^ [8].

### 3.4. Contamination Degree of House Dusts

**Figure 2:**
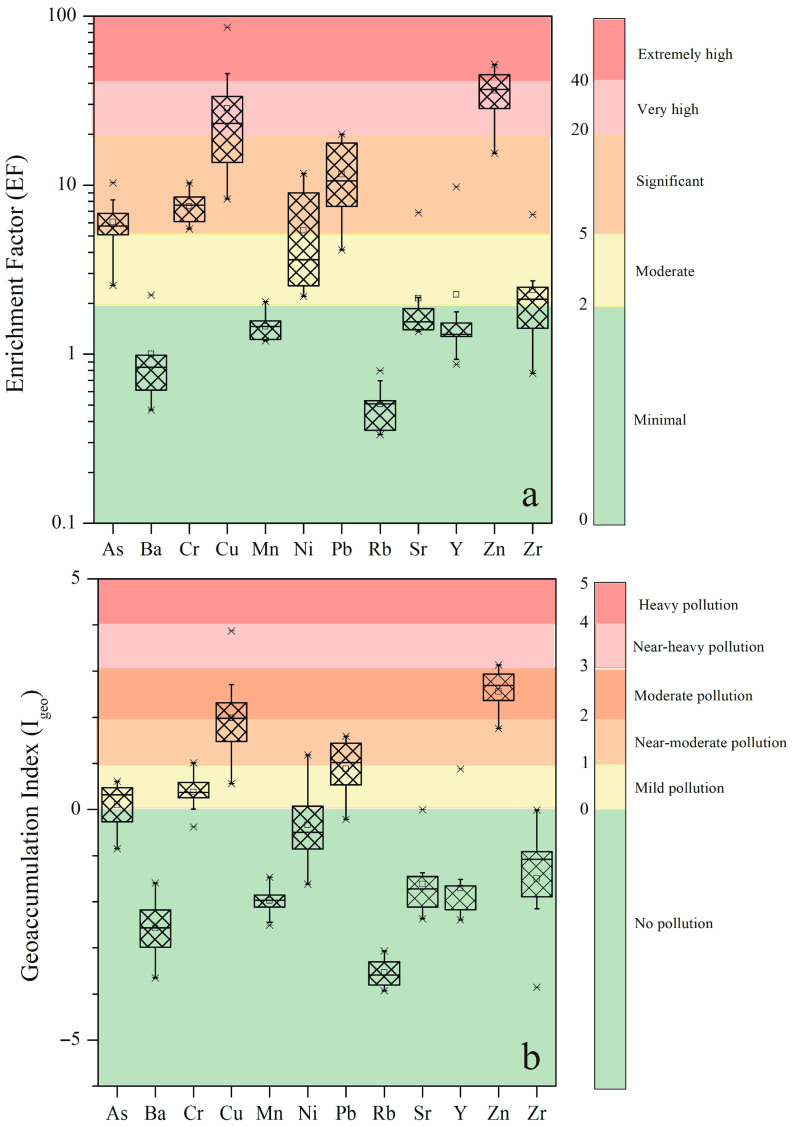
Enrichment factor (EF, (**a**)) and Geoaccumulation index (I_geo_, (**b**)) calculations (mean) for the assessed trace elements. Shaded categories indicate the level of enrichment and pollution, respectively.

To assess the impact of anthropogenic activities on the levels of PTEs in household dusts, EF and I_geo_ were computed, and their values are presented in Figure 2. The mean EFs followed a descending pattern of Zn > Cu > Pb > Cr > As > Ni > Zr > Y > Sr > Mn > Ba > Rb. Zinc and Cu returned values EF > 10 in all sampled house dusts, while EFs for Pb were above 10 in more than 50% of the sampled residences, in line with previous studies for indoor environments [78]. Specifically, the median EF values of Zn (36.8), Cu (23.2), and Pb (10.6) were higher than 10, implying that these elements in house dusts are related to anthropogenic and/or household activities [46]. On the contrary, the median EFs for Cr (7.6), As (5.7), and Ni (3.6) indicate a moderate enrichment linked mainly to the lithogenic component of the house dust, mainly inherited from the outdoor soil; minor anthropogenic loads could not be excluded. EF values did not exhibit great variability among sampled house dusts, suggesting the absence of local anthropogenic activities adjacent to the sampled residential environments. However, residences close to the city center presented significantly higher EF values reflecting possible outdoor inputs from adjacent roadside environments, as enhanced heavy metal contents in road dusts have already been reported [86].

Similar to EFs, the mean I_geo_ values decreased in the order of Zn > Cu > Pb > Cr > As > Ni > Zr > Y > Sr > Mn > Ba > Rb (Figure 2). The majority of the sampled house dusts are characterized as “unpolluted” with regard to Ba, Mn, Rb, Sr, Y, and Zr. On the contrary, Zn and Cu emerged as the most significant contaminants, with 20% of the sampled house dusts categorized as “near-heavily polluted”, while almost 50% of the house dusts were characterized as “moderately polluted”. In comparison, Pb and Cr showed moderate pollution levels, pointing to a notable anthropogenic impact. In particular, for Pb, 50% of the sampled dusts were rated as “near-moderate polluted”, while with regard to Cr, 80% of the house dusts were categorized as “mildly polluted” with an additional 10% considered as “near-moderate polluted”.

### 3.5. Source Identification

In order to elucidate the potential sources of PTEs identified in the house dusts, Principal Component Analysis (PCA) was applied to the complete dataset. The PCA results, including component loadings, percentage of variance, and total eigenvalues of the principal components (PCs), are summarized in Table 3. In total, six PCs were identified, accounting 93% of the total variance, with the most significant loadings discussed below.

PC1, explaining 20.5% of the variance, is characterized by very high loadings for Ca (0.966), Sr (0.956), Al (0.730), and a moderate loading for Ba (0.538) representing a dominant construction material source likely reflecting the influence of building-derived input (e.g., cement, concrete dust, or soil resuspension) due to deterioration of building material and the composition of wall materials. The relatively high loading for Ca and Sr indicates their common origin in indoor construction carbonate-based materials such as calcite and gypsum, which are key components of plaster, cement, mortar, and other construction materials frequently used in residential buildings. The degradation, abrasion, or renovation of these materials releases particulate matter rich in Ca and associated Sr into the indoor environment. This relationship has been consistently reported in the literature, where Ca–Sr correlations in indoor dust have been used as tracers for building-derived inputs [42,66]. Rasmussen et al. [42] underscored that the co-enrichment of Ca and Sr in settled dust indicates the contribution from plaster and wall materials, while similar associations were highlighted by Dingle et al. [87] in studies differentiating indoor from outdoor dust sources. The persistence of such patterns across multiple indoor environments supports the interpretation that the co-existence of Ca–Sr acts as a solid clue for construction material inputs to household dust. In addition, weak and insignificant negative loadings for several PTEs were recorded, which are consistent with a dilution effect towards Ca-rich particles [88].

PC2 explaining 17.7% of the variance, exhibits strong associations with Fe (0.959), Ti (0.832), Mn (0.824), and Cr (0.706), reflecting lithogenic inputs from adjacent soils that infiltrate indoor environments as seen across diverse studies [80,89]. Nonetheless, stainless-steel components within homes composed primarily of Fe, Cr, and often Mn and Ti [90] could contribute to indoor dust loads. Wear, corrosion, or abrasion of such materials (e.g., cooking appliances, fixtures, hardware) may release metal particles, complicating source apportionment. This potential is supported by geochemical studies that separate Fe-Cr clusters from crustal element clusters, implying alternative anthropogenic sources [91], as well as factors correlating Cr with building materials [92], paints [93], and carpet dyes [8,66]. Therefore, although crustal inputs dominate, the contribution from alternative indoor sources cannot be ruled out.

PC3 explaining 16.2% of the variance, is notably marked by high loadings for Zn (0.860), Pb (0.818), As (0.781), and moderate loadings for Cu (0.649) and Zr (0.418), implying strong anthropogenic contributions widely documented in indoor and soil environments [33,66,94]. This is remarkably consistent with the “Pb-Zb-As” cluster identified by Isley et al. [8] in residential indoor dusts from 35 countries, which was linked to legacy uses of Pb-containing materials (e.g., paint, plumbing, solder), home age, and urban emissions. The joint presence of Cu and Zr reinforces a traffic-related or wear-derived contribution as Cu often originates from brake pad abrasion [72], while Zr is associated with both vehicular wear [95,96,97] and certain construction materials [98].

PC4 (14.2% of variance) displays strong contributions from Si (0.846) and Ni (0.811) and a moderate loading for Cu (0.508), Al (0.574), pointing to a mixed origin component, predominantly a crustal or outdoor source, along with a superimposed anthropogenic signal from Ni and Cu sources. Si is typically indicative of soil or mineral dust, suggesting a geogenic source transported indoors via resuspension, shoes, or open windows. On the contrary, Ni is more often linked to anthropogenic sources such as fuel oil, combustion, stainless-steel wear, or industrial emissions, although some natural contribution is possible from ultramafic rocks [8]. The co-loading of Cu adds further weight to an anthropogenic influence tied to break wear, domestic plumbing, or electrical components [8,66]. Moreover, the Si-Ni-Cu component may also reflect contributions from metal alloys extensively used in electronic Pb frames for integrated circuits [99]. Thus, aging or degradation, especially during machining, abrasion, or wear, may lead to the release of fine particles enriched in Si, Ni, and Cu into indoor environments.

PC5 (12.5% of variance) is dominated by K (0.729), followed by Zr (0.576) and Rb (0.482), likely representing a crustal source originating from weathered silicate minerals such as feldspars and micas. The strong negative loading for Y (−0.915) suggests a distinct, non-crustal source.

Finally, PC6 exhibits a single strong negative loading of Mg (−0.963), which may reflect a distinct compositional pathway or unique geochemical behavior.

**Figure 3:**
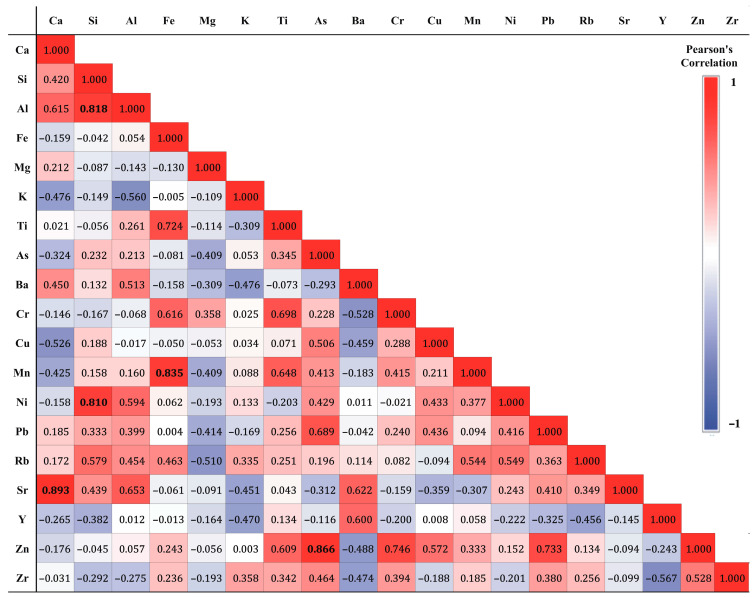
Correlation matrix representing Pearson’s correlation coefficients among the assessed trace elements. Values in bold represent strong correlation.

The PCA results were further supported by Pearson’s correlation matrix (Figure 3), which digs a little deeper into how the elements relate and where they might come from. Ca and Sr, for example, show a strong positive correlation (r = 0.893), which was not surprising as their geochemical association is well-established. Strontium often substitutes calcium in the crystal lattice of carbonate minerals because of their similar ionic radii and charge [100], resulting in parallel variations in environmental samples. In indoor environments, this pairing usually points to the presence of carbonate-rich building materials, all of which tend to release both elements into the environment [42]. Fe exhibited a strong correlation with Mn (r = 0.835), Ti (r = 0.724), and Cr (r = 0.616), indicating a common geogenic source, possibly related to resuspended outdoor dust. Cr presented a moderate positive correlation with Ti (r = 0.698) and Mn (r = 0.415), supporting its association with natural mineral inputs, although relationships among Fe, Cr, Ti, and Mn may also reflect mixed origins, including potential contributions from indoor sources, like metal surfaces, paints, or building materials. As for the other elements, As and Zn had a strong correlation (r = 0.866), as did Pb and Zn (0.733), and As and Pb (r = 0.689), underlying their common anthropogenic input into household dusts. Finally, Al, Si, and Ni show a strong connection (r > 0.81), which likely points to a shared lithogenic source; the moderate correlation between Cu and Ni (r = 0.433) signals some anthropogenic contributions, as already discussed. These patterns between specific elements reinforce the factor structures from the PCA and further validate the identification of both natural and anthropogenic inputs in the indoor dust samples.

### 3.6. Health Risk Assessment

**Figure 4:**
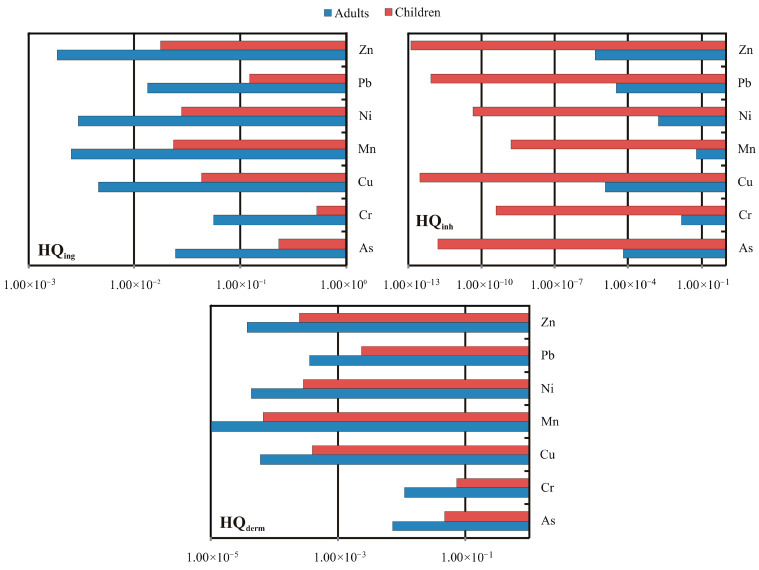
Average non-carcinogenic hazard quotients (HQs) of exposure through ingestion (HQ_ing_), inhalation (HQ_inh_), and dermal contact (HQ_derm_) to the assessed trace elements in house dusts from the city of Thessaloniki.

The potential non-carcinogenic and carcinogenic health risks associated with exposure to PTEs in house dust were evaluated for each sampled house dust, for both adults and children, via three exposure pathways: ingestion, inhalation, and dermal contact. The calculated Hazard Quotient (HQ), Hazard Index (HI), and Cancer Risk (CR) values are summarized in Table 4, while the average HQs through all exposure pathways are illustrated in Figure 4. The results demonstrated clear age-related differences in vulnerability, with children consistently exhibiting higher HQs across all evaluated elements, which is in agreement with previous studies on indoor dust exposure [8,101,102]. 

Among the three exposure pathways, ingestion was the dominant exposure route for both adults and children, as evidenced by the significantly higher HQ_ing_ values (Table 4). Specifically, for non-carcinogenic risk, ingestion exposure yielded the highest average HQs in both age groups, following a descending order of Cr > As > Pb > Cu > Ni > Mn > Zn for both adults and children. Cr and As exhibited the greatest non-carcinogenic threat for children, with mean HQ_ing_ values of 5.19 × 10^−1^ and 2.27 × 10^−1^, respectively, both approaching the risk threshold of 1, suggesting elevated potential for health impacts, particularly in more contaminated indoor environments. Similarly, Pb also presented a relatively high risk for children (1.23 × 10^−1^) approaching the unity, suggesting an elevated potential for adverse health effects. For adults, the HQ values through ingestion were significantly below the level of concern, varying from 1.87 × 10^−3^ (Zn) to 5.56 × 10^−2^ (Cr), suggesting a minimal likelihood of non-carcinogenic health effects via this exposure route. Moreover, children exhibited significantly elevated cumulative ingestion risks, with values ranging from 4.44 × 10^−1^ to a peak of 1.30, underscoring their vulnerability to heavy metal exposure via unintentional ingestion of house dust, with maximum values exceeding the threshold of 1, thus implying potential adverse health effects in high-exposure scenarios.

Dermal contact was the second most important exposure route. The average HQ_derm_ values followed the trend Cr > As > Pb > Cu > Ni > Zn > Mn for both adults and children. Chromium again emerged as the leading contributor to dermal exposure risk, with mean values of 1.11 × 10^−2^ (adults) and 7.27 × 10^−2^ (children), followed by As and Pb. Although these values remained below the critical threshold of 1, the relatively high values for Cr, particularly in children, suggest a need for continued monitoring and potential intervention. Inhalation exposure contributed negligibly to total non-carcinogenic risk in both age groups, with values several orders of magnitude lower than ingestion or dermal contact.

Cumulative non-carcinogenic risks, expressed as the hazard index (HI), also supported this trend. For children, mean HI values ranged from 1.80 × 10^−2^ (Zn) to 6.20 × 10^−1^ (Cr), reflecting moderate concern for adverse health effects in high-exposure scenarios. In contrast, adult HI values were lower, ranging from 1.94 × 10^−3^ (Zn) to 8.51 × 10^−2^ (Cr), with maximum values not exceeding 1.22 × 10^−1^ (for Cr), thus indicating negligible non-carcinogenic risk from indoor dust exposure under current assumptions. Although all values remained below the critical threshold of 1, the upper range approached it, suggesting that in households with higher contamination, children may be at greater risk of adverse health effects due to their increased sensitivity and exposure rates. Moreover, though individual HIs for most metals remained below the threshold of concern, their combined impact contributed to elevated total HI values, particularly in children (1.04). This cumulative burden reflects the potential for additive or even synergistic toxicological effects, which may increase the likelihood of adverse health outcomes even when individual metals do not pose significant risk on their own. Consequently, health risk assessments should emphasize integrated exposure to mixtures of contaminants, as real-world exposures rarely occur in isolation.

Carcinogenic risk (CR) was assessed for As, Cr, and Ni. Among these, Cr exhibited the highest cancer risk values for both adults and children (Table 4). For adults, CR ranged from 2.49 × 10^−5^ to 6.55 × 10^−5^ (mean: 4.35 × 10^−5^), not exceeding the acceptable risk range of 10^−6^ to 10^−4^. Arsenic (As) also presented CR values slightly above acceptable levels (mean: 5.71 × 10^−6^), while CR for Ni (2.60 × 10^−5^) remained within tolerable limits but warrants consideration due to cumulative effects.

## 4. Discussion

Research on potentially toxic elements (PTEs) in household dust has been ongoing for almost five decades. Some of the earliest investigations, such as those by Solomon and Hartford [39] in the United States and Harrison [103] in the United Kingdom, concentrated on detecting Pb and Cd in residential environments. The reported PTE concentrations in household dust from Greece were comparable to those in other regions worldwide (Table 5). The elemental profile of HDs followed an almost consistent pattern, with concentrations decreasing in the order Zn > Mn > Cu (Table 2). This trend aligns with prior studies from Volos [46] and Athens [44,45]. In Thessaloniki, Zn concentrations (650 mg kg^−1^) were greater than those reported in Athens (401 mg kg^−1^) and comparable to the corresponding ones in Volos (785 mg kg^−1^). Nickel concentrations in Thessaloniki (50 mg kg^−1^) were also slightly elevated in relation to the corresponding ones in Athens [44,45]. Finally, Pb concentrations in HDs from the city of Thessaloniki (52 mg kg^−1^) were marginally higher than the recently reported values (46.1 mg kg^−1^) by Stamatelopoulou et al. [45], but slightly lower than the corresponding ones reported for Athens in a previous study (92.2 mg kg^−1^) by Kelepertzis et al. [44]. Industrial areas exhibited relatively higher Pb contents, with values reported as high as 4000 mg kg^−1^ in Lavrion [104], while in Stratoni reached 1660 mg kg^-1^ [47], underscoring the significant impact of former mining and industrial operations in the areas.

On a global scale, Cu concentrations in Thessaloniki (166 mg kg^−1^) were comparable to those reported in Australia (186 mg kg^−1^), Croatia (133 mg kg^−1^), and China (119.9 mg kg^−1^), in contrast to significantly higher levels observed in Spain (965 mg kg^−1^) and the United Kingdom (301 mg kg^−1^). On the other hand, Zn levels in Thessaloniki (650 mg kg^−1^) did not exceeded Australia’s concentrations of 1260 mg/kg [105], UK’s (622 mg kg^−1^), Spain’s (883 mg kg^−1^) and surpassed values reported in Turkey (263 mg kg^−1^), Latvia (372 mg kg^−1^) and Ghana (114 mg kg^−1^). Ni levels were similar to those reported for Spain (55 mg kg^−1^), UK (53.1 mg kg^−1^), and China (51.7 mg kg^−1^), while they were lower than those reported for Jordan (126.4 mg kg^−1^), following Canada (62.4 mg kg^−1^). The presence of Ni in indoor environments is typically linked to the degradation of metal components, building materials, and ambient contributions from traffic and urban emissions. Regarding Pb levels in sampled house dusts, the recorded mean values (52 mg kg^−1^) were considered moderate in a global context. Specifically, there were lower than those reported worldwide, except for a few countries such as Latvia (21.4 mg kg^−1^), Turkey (27.5 mg kg^−1^), and South Korea (29 mg kg^−1^). Nevertheless, Pb levels in Thessaloniki remain significantly lower than those recorded in cities such as Spain (152 mg kg^−1^), the United Kingdom (150 mg kg^−1^), and Canada (100 mg kg^−1^), reflecting comparatively reduced historical or industrial Pb inputs.

Of particular importance are the elevated Cr and Mn contents observed in the studied household dusts compared to those reported in previous studies conducted both within Greece and globally (Table 5), as summarized in Figure S1. The mean Cr (179 mg kg^−1^) levels surpass those documented in other urban environments across Europe [8,33,106], Asia [66,107,108,109], and America [110], highlighting a notable deviation from global literature data. Similarly, mean Mn concentrations (384 mg kg^−1^) were among the highest recorded in non-industrial urban settings globally, except for those in the United Kingdom (524 mg kg^−1^). The elevated Cr and Mn contents in house dusts are largely attributed to geogenic sources, particularly the influence of local geological substrates rich in ultramafic or mafic rocks [111,112]. Particularly, in Greece, elevated background levels of Cr and Mn have been reported in soils and sediments due to the presence of ophiolitic formations, which are naturally enriched in these elements [113,114]. These geogenic signatures can be transferred indoors via soil resuspension, wind deposition, or foot traffic, contributing to the accumulation of Cr and Mn in settled house dust. Similar trends have been noted worldwide in regions where the parent materials are rich in metals. For instance, in indoor dusts from Canada and Tehran, respectively, Rasmussen et al. [51] and Khajooee et al. [80] found that natural sources were the main sources of Cr and Mn.

Furthermore, the persistently high levels of Cr and Mn also show a broader, worrisome pattern of their growing buildup in household dust over time. In particular, a comparison of Cr and Mn contents in household dusts from Thessaloniki based on preliminary (unpublished) data from our research team obtained through ongoing monitoring studies in 2021 to the corresponding ones in 2024 revealed a worrying upward trend for both elements, which should be interpreted with caution. Specifically, Cr levels increased from 125.6 mg kg^−1^ in 2021 to 179 mg kg^−1^ in 2024, while Mn concentrations rose from 251 mg kg^−1^ to 384 mg kg^−1^ over the same period. In addition to geogenic background, these notable increases in a comparatively short period of time point to an increasing accumulation of Cr and Mn in the indoor environment, which may reflect superimposed anthropogenic contributions. These could be caused by widespread use of Cr/Mn-containing materials, especially in the transportation and construction industries, or by possible localized geogenic/anthropogenic influences. Moreover, the observed increase suggests that either sources of contamination have surfaced or that current environmental controls are insufficient to reduce Cr and Mn emissions. In any case, this trend should be interpreted with caution as it is based on temporal data.

**Table 5:** Mean concentrations (mg kg^−1^) of potentially toxic elements (PTEs) in house dusts collected from various cities around the world.

Study Areas	n ^1^	As	Cr	Cu	Mn	Ni	Pb	Zn	References
Thessaloniki, Greece	20	9	179	166	384	50	52	650	This study
Athens, Greece	43		82.9	217	132	89.5	92.2	786	[44]
Athens, Greece	20	4	65.2	339	128	29.9	46.1	401	[45]
Volos, Greece	24	31.7	163	167	274	81	63.2	785	[46]
Stratoni, Greece	30			446	1250		1660	2720	[47]
Lavrio, Attica	127						4000		[104]
Croatia	34	4.5	88.5	133	225	32.5	59	669	[8]
Ghana	54	1.75	51	55.5	161.5	17	60.5	114	[8]
Spain	56	22	69	965		55	152	883	[33]
Turkey	85	4.41	23.8	65.7	65.9	32.3	27.5	263	[66]
Australia	1310	19	86	186	246	36	126	1260	[105]
Lithouania	120	0.78	41.6	69.3	74.5	12.4	21.4	372	[106]
Jordan	70		36.2	94.8	141.3	126.4	83.4	531.7	[107]
Korea	106	2.2	48		105	44	29		[108]
China	21	9.8	66.4	119.9	273	51.7	87.4	442.2	[109]
Canada	1025	9	99			62.4	100		[110]
United Kingdom	32			301	524	53.1	150	622	[115]
DustSafe (Greece dataset)	35	6.9	97.9	158	230	52.3	57	664	[8]
DustSafe (means of 35 countries)	2265	13.3	86	176	257	39	94	1110	[8]

^1^ number of dust samples analyzed.

The rise in Cr levels brings real worries about health risks associated with chronic exposure, especially since certain forms of Cr, such as Cr(VI), are known to be toxic and carcinogenic. Our results show that children face greater danger from both non-carcinogenic and carcinogenic risks linked to indoor dust exposure, mainly because of Cr presence. Similar high Cr-related health risks have been reported in Iranian cities [79] and multiple provinces around China [116]. Furthermore, a recent study in India [117] reported that children’s HI values, although generally below 1, were significantly higher than adults’, with Cr identified as the dominant contributor to the cumulative non-carcinogenic risk due to its elevated toxicity and high exposure potential in indoor environments. In Hefei, China, children were found to have higher hazard index (HI) values than adults, even though most values remained below the threshold of 1. Ingestion was identified as the primary exposure route [34]. On the other hand, results in Kuwait were more concerning, with both children and adults recording HI values above 1, with Cr accounting for 65–68% of total HI [3]. On a broader scale, data from 35 countries showed that non-carcinogenic HIs for children were generally within acceptable limits (<1), Cr consistently emerged as the dominant contributor to overall risk [8]. All the aforementioned findings suggest that while individual HQs may not always be alarming, their cumulative impact (particularly through ingestion in children) can increase overall risk. Excessive Cr exposure has been related to plausible neurological problems, improper blood coagulation, and harm to the human body’s vital organs, such as the liver and kidneys [50].

In advance, potential health effects are strongly influenced by the mineralogy and morphology of indoor dust particles. Quartz, which is a form of crystalline silica commonly found in household dust, has been linked to serious lung disorders, especially when it is present in the respirable fraction (<4 μm) [27,28,118]. Although the <150 μm size fraction utilized in the present study was not representative of the respirable fraction, it constitutes a reservoir of finer particles. Therefore, dominance and invariable presence of quartz in all dust samples (Table 1) may be considered as a potential for respiratory exposure which might trigger lung inflammation and even long-term respiratory diseases, with toxicity often enhanced by the small particle size and high surface area of dust particles [119]. In addition, the mineralogical profile of sampled dusts, presenting a major calcite phase and minor clay abundances, may critically dictate the potential health risks. While the dominance of calcite acts as a geochemical diluent, resulting in underestimation of risk [88], clay minerals function in the opposite manner by acting as a powerful geochemical sink due to their high specific surface area and their net negative surface charge [120,121]. Also, physical characteristics such as shape, sharpness, and roughness of dust particles really matter when it comes to how they act in the respiratory tract, with irregular or sharp-edged particles being able to actually irritate the respiratory tissues [122]. The predominance of Fe/Cr particles in the indoor dust samples (Figure 1), pointed to both natural and anthropogenic sources mixing together, while the irregular morphology, often angular or sharp-edged, was linked with mechanical abrasion of stainless-steel components, household appliances, or construction materials [30,31], as well as resuspended soil particles containing Fe and Cr [111]. This particle shape matters for human health, since irregular and angular particles slip deeper into the lungs and cause more tissue irritation or oxidative stress than spherical ones [122]. The predominance of irregular and quartz-containing particles underlines the need for integrated exposure assessments that account not only for the elemental composition but also the mineralogy and morphology of indoor dust to fully capture and evaluate the potential health hazards posed by indoor dust.

## 5. Conclusions

House dust represents a complex and often underestimated source of environmental contaminants with potentially significant health implications. This study provides a comprehensive characterization of residential house dust from the city of Thessaloniki, Greece, integrating chemical, mineralogical, morphological, and health risk analyses. The crystalline fraction was dominated by mineral phases such as calcite, quartz, and plagioclase, while morphological examination revealed particles with angular, irregular, and spherical shapes. Aluminosilicates, along with Ca- and Fe-rich particles, originated from both outdoor (traffic and soil) and indoor sources (e.g., stainless steel components, soldering materials).

Indoor dust acts as a sink for both geogenic and anthropogenic contaminants, with elevated concentrations of Zn, Cu, Pb, Cr, and Mn. Of particular concern were the high levels of Cr and Mn, among the highest reported for non-industrial indoor environments, which are attributed to local geology and reinforced by human activities. Temporal comparison indicates a concerning upward trend in Cr and Mn contents, warranting further monitoring.

Multivariate analysis identified distinct sources: (i) Ca–Sr from construction materials, (ii) Fe–Cr–Mn–Ti from lithogenic and stainless steel sources, (iii) Zn–Pb–As–Cu from anthropogenic legacy materials, (iv) Si–Ni–Cu from alloy-related sources, and (v) K–Rb–Zr from aluminosilicate minerals.

Health risk assessment showed that children are the most vulnerable group, with ingestion being the most critical exposure pathway. Chromium and As contributed the highest to both non-carcinogenic and carcinogenic risks, with hazard indices approaching the safe limit of 1, while cancer risk estimates for Cr did not exceed USEPA thresholds.

Overall, this study highlights the complex origin of house dust and its role as a carrier of PTEs. The findings underscore the need for continued monitoring, along with the development of effective interventions to reduce exposure, particularly in households with children or other vulnerable individuals.

## Figures and Tables

**Table 1:** Mineralogical composition (in % *w*/*w*) of house dusts from Thessaloniki city estimated by XRD. Key: Cc-Calcite, Qz-Quartz, Dol-Dolomite, Pl-Plagioclase, Mc-Micas, Chl ± Kaol-Chlorite ± Kaolinite, Tc-Talc.

	Cc	Qz	Dol	Pl	Μc	Chl ± Kaol	Tc	Swelling Clays
Number of samples	20	20	20	18	18	14	16	8
Min	27	12	2	4	3	1	2	2
Max	60	31	13	14	10	8	8	7
Mean	46	19	6	8	6	4	4	5
SD ^1^	9	6	3	4	3	2	2	2

^1^ standard deviation.

**Table 3:** Main group components derived by principal component analysis (PCA). Values in bold represent strong correlation in specific components, while values underlined indicate moderate correlation.

	1	2	3	4	5	6
Ca	**0.966**	−0.117	−0.103	−0.071	0.087	−0.159
Si	0.481	−0.036	0.002	**0.846**	0.182	0.040
Al	**0.730**	0.128	0.082	0.574	−0.199	0.108
Fe	−0.032	**0.959**	−0.060	0.021	0.002	0.097
Mg	0.032	−0.042	−0.135	−0.057	0.047	**−0.963**
K	−0.529	−0.038	−0.142	−0.030	**0.729**	0.161
Ti	0.162	**0.832**	0.390	−0.130	−0.171	−0.020
As	−0.113	0.346	**0.781**	0.152	0.061	0.392
Ba	0.538	0.270	−0.075	−0.190	−0.499	0.479
Cr	−0.086	**0.706**	0.478	−0.083	0.098	−0.413
Cu	−0.434	−0.080	0.649	0.508	−0.173	−0.112
Mn	−0.263	**0.824**	0.047	0.338	−0.012	0.324
Ni	−0.390	0.162	0.058	**0.811**	0.146	0.130
Pb	0.343	−0.013	**0.818**	0.187	0.104	0.352
Rb	0.314	0.429	−0.046	0.394	0.482	0.516
Sr	**0.956**	−0.090	0.005	−0.006	−0.038	0.137
Y	−0.161	0.111	−0.105	−0.181	**−0.915**	0.199
Zn	−0.080	0.169	**0.860**	−0.275	0.043	−0.247
Zr	−0.045	0.301	0.418	−0.490	0.576	0.240
Eigenvalue	3.889	3.372	3.079	2.707	2.373	2.257
Explained variance (%)	20.5	17.7	16.2	14.2	12.5	11.9
Cumulative (%) of variance	20.5	38.2	54.4	68.7	81.2	93.0

**Table 4:** Non-carcinogenic and carcinogenic risks of each element and exposure pathway for house dusts from the city of Thessaloniki, Greece. Values underlined indicate values approaching the risk threshold of 1.

		Adults				Children					CR
		HQ_ing_	HQ_inh_	HQ_derm_	HI	HQ_ing_	HQ_inh_	HQ_derm_	HI		
As	Min	1.20 × 10^−2^	2.98 × 10^−5^	3.50 × 10^−3^	1.55 × 10^−2^	1.12 × 10^−1^	8.17 × 10^−13^	2.30 × 10^−2^	1.35 × 10^−1^	As_carc_	2.82 × 10^−6^
	Max	3.30 × 10^−2^	8.19 × 10^−5^	9.64 × 10^−3^	4.27 × 10^−2^	3.08 × 10^−1^	2.25 × 10^−12^	6.31 × 10^−2^	3.71 × 10^−1^		7.74 × 10^−6^
	Mean	2.43 × 10^−2^	6.04 × 10^−5^	7.11 × 10^−3^	3.15 × 10^−2^	2.27 × 10^−1^	1.66 × 10^−12^	4.65 × 10^−2^	2.74 × 10^−1^		5.71 × 10^−6^
Cr	Min	3.18 × 10^−2^	8.28 × 10^−3^	6.35 × 10^−3^	6.09 × 10^−2^	2.97 × 10^−1^	2.27 × 10^−10^	4.16 × 10^−2^	4.44 × 10^−1^	Cr_carc_	2.49 × 10^−5^
	Max	8.37 × 10^−2^	2.18 × 10^−2^	1.67 × 10^−2^	1.22 × 10^−1^	7.81 × 10^−1^	5.97 × 10^−10^	1.09 × 10^−1^	8.91 × 10^−1^		6.55 × 10^−5^
	Mean	5.56 × 10^−2^	1.45 × 10^−2^	1.11 × 10^−2^	8.51 × 10^−2^	5.19 × 10^−1^	3.97 × 10^−10^	7.27 × 10^−2^	6.20 × 10^−1^		4.35 × 10^−5^
Cu	Min	1.40 × 10^−3^	3.46 × 10^−6^	1.86 × 10^−5^	1.42 × 10^−3^	1.30 × 10^−2^	9.49 × 10^−14^	1.22 × 10^−4^	1.31 × 10^−2^		
	Max	1.38 × 10^−2^	3.43 × 10^−5^	1.84 × 10^−4^	1.40 × 10^−2^	1.29 × 10^−1^	9.40 × 10^−13^	1.20 × 10^−3^	1.30 × 10^−1^		
	Mean	4.59 × 10^−3^	1.14 × 10^−5^	6.10 × 10^−5^	4.66 × 10^−3^	4.28 × 10^−2^	3.12 × 10^−13^	4.00 × 10^−4^	4.32 × 10^−2^		
Mn	Min	1.69 × 10^−3^	4.11 × 10^−2^	6.75 × 10^−6^	4.28 × 10^−2^	1.58 × 10^−2^	1.13 × 10^−9^	4.42 × 10^−5^	1.58 × 10^−2^		
	Max	3.50 × 10^−3^	8.50 × 10^−2^	1.40 × 10^−5^	8.85 × 10^−2^	3.26 × 10^−2^	2.33 × 10^−9^	9.14 × 10^−5^	3.27 × 10^−2^		
	Mean	2.52 × 10^−3^	6.12 × 10^−2^	1.00 × 10^−5^	6.37 × 10^−2^	2.35 × 10^−2^	1.68 × 10^−9^	6.58 × 10^−5^	2.36 × 10^−2^		
Ni	Min	1.04 × 10^−3^	5.71 × 10^−4^	1.53 × 10^−5^	1.62 × 10^−3^	9.66 × 10^−3^	1.56 × 10^−11^	1.00 × 10^−4^	9.76 × 10^−3^	Ni_carc_	9.07 × 10^−6^
	Max	7.20 × 10^−3^	3.97 × 10^−3^	1.06 × 10^−4^	1.13 × 10^−2^	6.72 × 10^−2^	1.09 × 10^−10^	6.97 × 10^−4^	6.79 × 10^−2^		6.31 × 10^−5^
	Mean	2.96 × 10^−3^	1.63 × 10^−3^	4.38 × 10^−5^	4.64 × 10^−3^	2.76 × 10^−2^	4.48 × 10^−11^	2.87 × 10^−4^	2.79 × 10^−2^		2.60 × 10^−5^
Pb	Min	5.66 × 10^−3^	1.40 × 10^−5^	1.51 × 10^−4^	5.82 × 10^−3^	5.28 × 10^−2^	3.85 × 10^−13^	9.86 × 10^−4^	5.38 × 10^−2^		
	Max	1.98 × 10^−2^	4.91 × 10^−5^	5.27 × 10^−4^	2.04 × 10^−2^	1.85 × 10^−1^	1.35 × 10^−12^	3.45 × 10^−3^	1.88 × 10^−1^		
	Mean	1.32 × 10^−2^	3.27 × 10^−5^	3.51 × 10^−4^	1.36 × 10^−2^	1.23 × 10^−1^	8.98 × 10^−13^	2.30 × 10^−3^	1.25 × 10^−1^		
Zn	Min	1.02 × 10^−3^	2.52 × 10^−6^	2.03 × 10^−5^	1.04 × 10^−3^	9.49 × 10^−3^	6.92 × 10^−14^	1.33 × 10^−4^	9.63 × 10^−3^		
	Max	2.66 × 10^−3^	6.59 × 10^−6^	5.30 × 10^−5^	2.72 × 10^−3^	2.48 × 10^−2^	1.81 × 10^−13^	3.47 × 10^−4^	2.51 × 10^−2^		
	Mean	1.87 × 10^−3^	4.64 × 10^−6^	3.73 × 10^−5^	1.94 × 10^−3^	1.74 × 10^−2^	1.27 × 10^−13^	2.44 × 10^−4^	1.80 × 10^−2^		

## Data Availability

The original contributions presented in this study are included in the article/Supplementary Materials. Further inquiries can be directed to the corresponding author.

## Supplementary Materials

The following supporting information can be downloaded at: https://www.mdpi.com/article/10.3390/toxics14040306/s1, Figure S1: Reported data on Cr and Mn contents for indoor dusts from various cities in Greece and around the world; Table S1: Percentage recoveries of certified reference materials (CRMs) obtained by pXRF. Shaded cells indicate values that are further than ±30% of the optimum (100%) in percentage CRM recovery analysis; Table S2: Limits of detection (LoD) of pXRF for analyzed elements; Table S3: Exposure parameters for different health risk scenarios; Table S4: Reference dose (*R_f_D*, in mg kg^−1^ d^−1^) and slope factor (*SF*, in mg kg^−1^ d^−1^) values for each metal and exposure pathway based on the USEPA Regional Screening Level (RSL) tables.
