# Supplementary material for: Occurrence of Potentially Toxic Elements (PTEs) Coupled with Mineralogical and Morphological Characteristics of Residential Indoor Vacuum Dusts from the City of Thessaloniki, Northern Greece
Source: Toxics. 2026 Mar 31;14(4):306. doi: 10.3390/toxics14040306 (PMC13120277; doi:10.3390/toxics14040306)
Supplement: Supplementary file 1 [file toxics-14-00306-s001.zip › toxics-4224359-supplementary.pdf]

Occurrence of potentially toxic elements (PTEs) coupled with mineralogical and morphological characteristics of residential indoor vacuum dusts from the city of Thessaloniki, Northern Greece

Kotsakostoudi Ch.[1], *Bourliva A.[2], Papadopoulou L.[1], Kantiranis N.[1]

[1] Department of Mineralogy-Petrology-Economic Geology, School of Geology, Aristotle University of Thessaloniki, 54124 Thessaloniki, Greece

[2] Soil Science Laboratory, School of Agriculture, Faculty of Agriculture, Forestry and Natural Environment, Aristotle University of Thessaloniki, 541 24Thessaloniki, Greece

Supplementary Materials

Table S1. Percentage recoveries of certified reference materials (CRMs) obtained by pXRF. Shaded cells indicate values that are further than ±30% of the optimum (100%) in percentage CRM recovery analysis.

CRM	JSI-1 Slate	JSO-1 Soil	JMS-1 Marine sediment	JSM-2 Marine sediment	SDO-1 Shale	NIST-1648a
**Ca**	103.0	101.4	87.4	89.3	93.7	
**Si**	103.3	101.1	97.5	93.2	103.3	93.3
**Al**	103.0	107.5	88.3	83.1	109.3	85.6
**Fe**	101.8	117.1	95.9	91.6	99.2	
**Mg**	93.6	60.8	79.6	104.1	88.7	91.9
**K**	106.5	94.0	90.9	84.6	102.5	94.1
**Ti**	101.2	126.6	98.1	93.3	99.5	
**As**						468.4
**Ba**	82.3	85.5	90.2	98.5	90.4	
**Co**	90.3	96.9	88.4	92.9	96.2	195.2
**Cr**	162.6	109.9	108.3	105.1	97	102.7
**Cu**	98	90.5	84.1	84.8	81.4	97.9
**Mn**	97.4	124.8	101.7	99.9	104.8	
**Ni**						151.7
**Pb**						113.5
**Rb**	86.3	131	90.9	100	86.5	111.8
**Sr**	94.8	107.1	93.5	90.7	98.5	100.9
**Y**	100	96.4	98.8	98	120.7	
**Zn**	79.6	74.3	75	70.5	76.4	107.9
**Zr**	112.6	105.2	150	123.6	127.9	

Table S2. Limits of detection (LoD) of pXRF for analyzed elements.

GEO Exploration (3 PHASE RESULTS)	Ca	SiO_2	Al_2O_3	Fe	MgO	K_2O	Ti	As	Ba	Cr
LOD (ppm)	33	N/A	1240	20	4100	55	40	2	42	17
Upper range (wt %)	31 %	100 %	69 %	67 %	70 %	15.3 %	7.1 %	2.3 %	10.7 %	3.7 %
**GEO Exploration (3 PHASE RESULTS)**	**Cu**	**Mn**	**Ni**	**Pb**	**Rb**	**Sr**	**Y**	**Zn**	**Zr**	
LOD (ppm)	4	11	5	4	1	1	1	2	3	
Upper range (wt %)	6.8 %	45.4 %	3 %	4.3 %	0.05 %	0.46 %	0.11 %	19 %	2.5 %	

Table S3. Exposure parameters for different health risk scenarios.

Parameters			Units	Children	Adults
**C**	Concentration		mg kg^{-1}		
**R_{ing}**	Ingestion rate		mg d^{-1}	200	100
**R_{inh}**	Inhalation rate		mg d^{-1}	20	7,60
**ET**	Exposure Time		h d^{-1}	24	24
**EF**	Exposure Frequency		d y^{-1}	365	365
**ED**	Exposure Duration		y	6	24
**BW**	Body weight		kg	15	70
**CF**	Conversion Factor			10^{-6}	
**AT**	Average Time	Non-Carcinogenic	d	2.19	8.76
		Carcinogenic	d	-	25.55
**PEF**	Particle Exposure Factor			1,36 10^{-9}	
**SA**	Surface Area		cm^2	2.8	5.7
**SAF**	Skin Adherence Factor		mg cm^{-2}	0,2	0,07
**ABS**	Dermal Absorption Factor			10^{-3}	

Table S4. Reference dose (R_fD, in mg kg^{-1} d^{-1}) and slope factor (*SF*, in mg kg^{-1} d^{-1}) values for each metal and exposure pathway based on the USEPA Regional Screening Level (RSL) tables.

**Element**	**R_fD**			**SF**		
	**Oral**	**Inhalation**	**Dermal contact**	**Oral**	**Inhalation**	**Dermal Contact**
**As**	3.00 × 10^{-4}		1.23 × 10^{-4}	1.5		3.66
**Cr**	3.00 × 10^{-3}	2.86 × 10^{-5}	6.00 × 10^{-5}	5.00 × 10^{-1}		
**Cu**	4.00 × 10^{-2}		1.20 × 10^{-2}			
**Mn**		1.40 × 10^{-1}				
**Ni**	2.00 × 10^{-2}	9.00 × 10^{-5}	5.40 × 10^{-3}	8.40 × 10^{-1}		
**Pb**	3.50 × 10^{-3}		5.25 × 10^{-4}			
**Zn**	3.00 × 10^{-1}		6.00 × 10^{-2}			

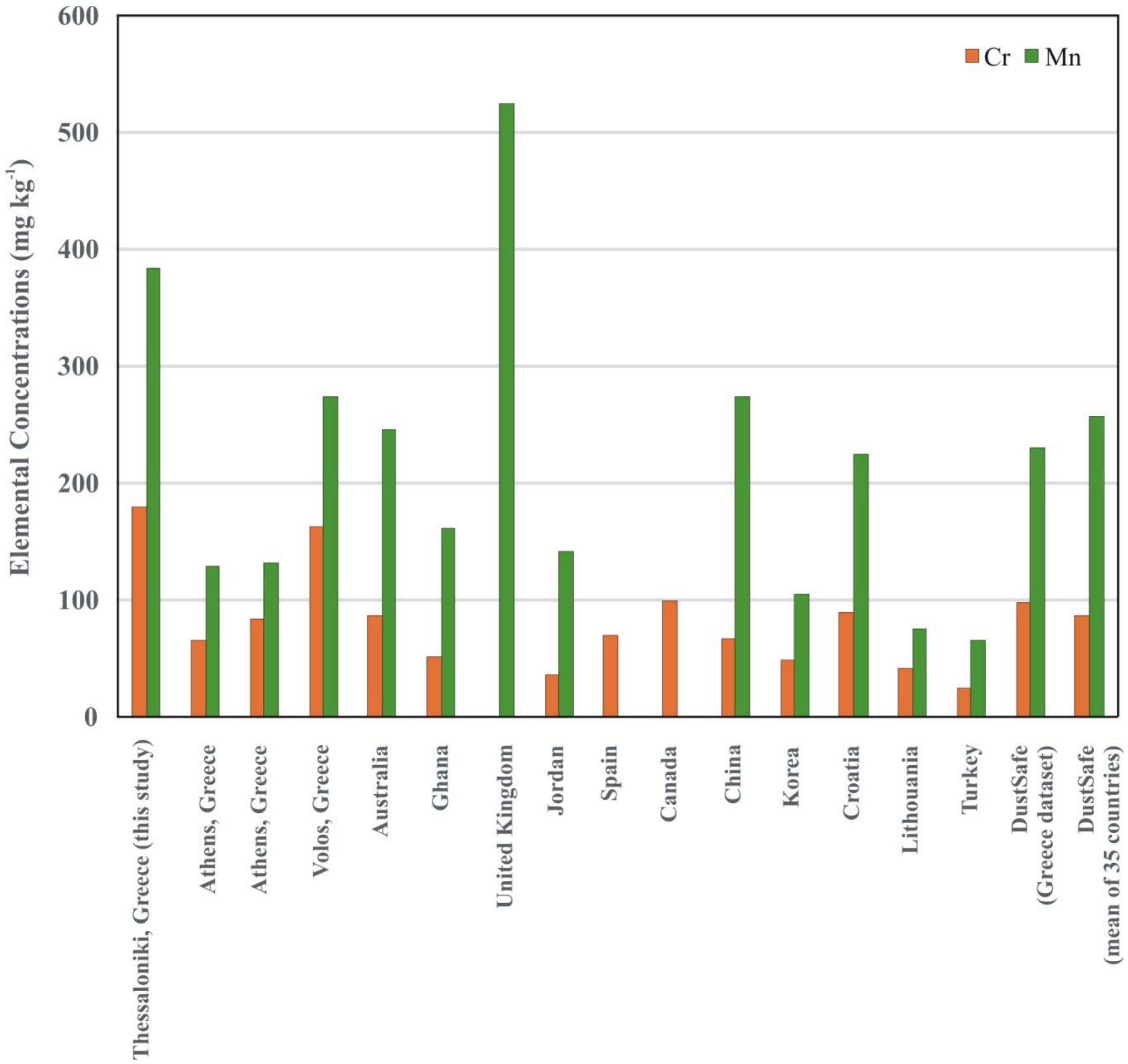

Figure S1. Reported data on Cr and Mn contents for indoor dusts from various cities in Greece and around the world.
